# Laparoscopic Nissen fundoplication in situs inversus totalis: Technical and ergonomic issues

**DOI:** 10.4103/0972-9941.72599

**Published:** 2010

**Authors:** Radha Govind Khandelwal, S Karthikeayan, T G Balachandar, Prasanna K Reddy

**Affiliations:** Department of Surgical Gastroenterology, Apollo Hospital, Chennai, India

**Keywords:** Laparoscopy, fundoplicaiton, situs inversus totalis

## Abstract

We report a laparoscopic Nissen fundoplication for gastroesophageal reflux disease (GERD) in a patient with situs inversus totalis (SIT). A 34-year-old man was diagnosed with SIT on performing chest X-ray and abdominal sonography as a routine preoperative investigations. He presented with chronic gastro-esophageal reflux disease (GERD) inadequately controlled by medications. The laparoscopic procedure was performed using five ports placed in a mirror-image configuration and with the patient in the modified lithotomy position. Few technical difficulties were encountered during the operation. The position of the primary surgeon, working between the lower limbs of the patient as in case of standard fundoplication, was considered most prudent position to the success of this case. In SIT, this position provides the least visual disorientation from the reversed abdominal organs. We recommend that preoperative detection of SIT is essential to understand the symptomatology of the patient and for planning of any upper abdominal laparoscopic procedure.

## INTRODUCTION

Laparoscopic Nissen fundoplication has almost totally replaced the open procedure. As the number of laparoscopic procedures continues to increase, it is inevitable that more patients with congenital anatomic anomalies will be subjected to laparoscopic surgery.

SIT has a reported incidence of 0.01% in the United States of America,[1] making this positional anomaly very uncommon. It is characterized by transposition of abdominal viscera, and when associated with a dextrocardia it is referred to as situs inversus totalis (SIT). Yet because this arrangement, called situs inversus, is a perfect mirror image, the relationship between the organs is not changed, so functional problems rarely occur. Generally, patients with SIT are asymptomatic and have a normal life expectancy.[2]

Documenting situs inversus in an individual is important in order to correctly interpret any future symptoms, to modify the surgical technique appropriately in order to prevent any inadvertent clinical or surgical mishap.

We present what is possibly the first reported case of laparoscopic Nissen fundoplication in a patient with SIT from India.

## CASE REPORT

**Figure 1:**
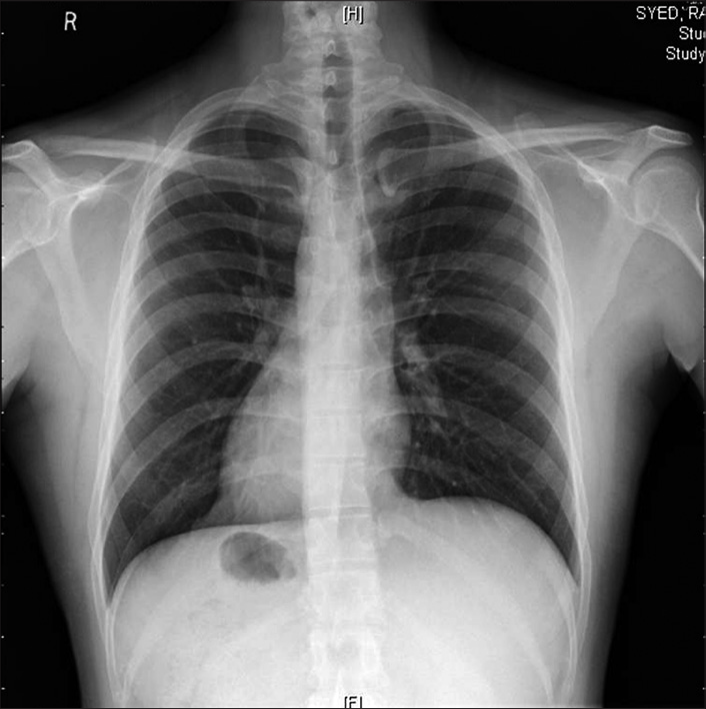
X-ray chest showing dextrocardia and gastric air bubble on right side i.e. situs inversus totalis.

**Figure 2:**
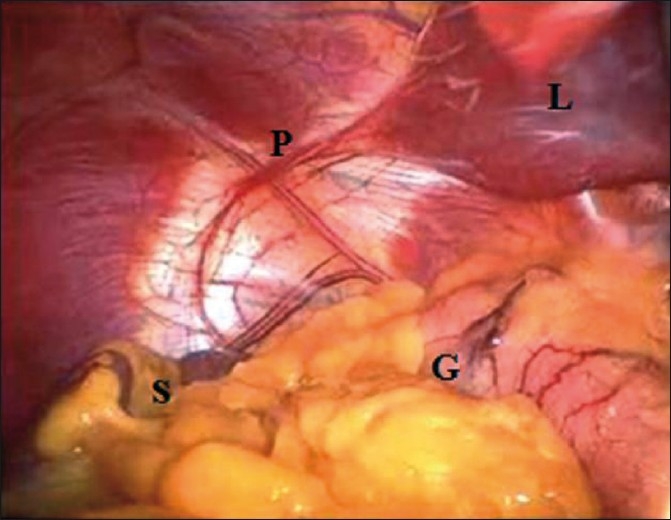
Mirror image of intraabdominal organs in situs inversus totalis (endoscopic view). G- greature curvature of stomach, S- spleen, P- Pericardium, L– Liver lobe.

A 34-year-old man presented with a 3-year history of symptoms consistent with GERD. His symptoms were initially controlled with proton-pump inhibitors but deteriorated in last 6 months. A chest X-ray detected SIT [Figure 1]. Oesophago-gastroduodenoscopy confirmed reflux oesophagitis with a lax lower oesophageal sphincter and sliding hiatal hernia. Patient did not cooperate for oesophageal manometry and 24 hour pH study. Nuclear gastroesophageal reflux study showed grade III reflux and hiatus hernia. The patient was offered a Nissen fundoplication. This was caried out with the patient in modified lithotomy position, which is the position we prefer for all cases of laparoscopic Nissen fundoplication. The ports were placed in a configuration that was the mirror image of our usual fundoplication procedure. One 10-mm supraumbilical camera port, 5-mm epigastric port for a Nathanson’s retractor, one 10-mm left midclavicular port and two 5-mm accessory ports below the right subcostal margin were placed. The surgeon stood in between the legs of the patient. The assistant worked from the right side of the patient, opposite his usual position. The intra-abdominal organs were visualized and SIT was confirmed [Figure 2].

**Figure 3:**
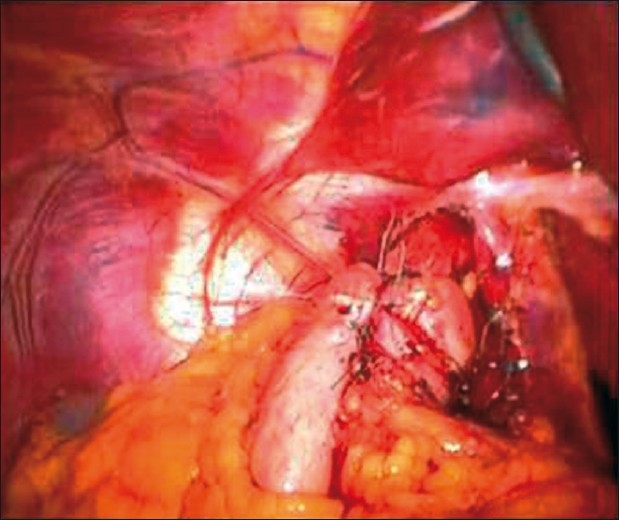
Endoscopic view showing completed Nissen fundoplication and cruroplasty.

The procedure was carried out in the standard fashion with intrathoracic dissection of the oesophagus and the cardia of the stomach, which was brought down below the oesophageal hiatus with adequate length and without tension. The crura were identified and dissected, and short gastric vessels were divided with an ultrasonic shears using the flat blade to mobilise the gastric fundus. Closure of the crura and a floppy Nissen fundoplication were performed with 2/0 polyester (Ethibind, Johnson and Johnson, Mumbai, India) sutures tied intra-corporeally [Figure 3]. The total operative time was 110 minutes. The patient was discharged on first postoperative day after he tolerated oral liquids.

## DISCUSSION

The majority of reports of laparoscopic procedures in patients with SIT cite technical difficulties and longer operative times due to disorientation from the reversed abdominal organs and the modification of the surgeon’s movements and techniques.[3–6]

However, with the patient in the modified lithotomy position and the primary surgeon working in between the legs, fundoplication could be performed with ease and without the difficulties in orientation. This may be because the oesophagus, oesophageal hiatus and stomach are all midline structures and so do not cause as much disorientation as does the gallbladder. More importantly, operating in the lithotomy position may have decreased the sensation of disorientation.[7]

At our centre we perform LNF with the primary surgeon in between the legs of patient even in patients with normal anatomy. So we followed the same positioning of the surgeon. We placed the port as the mirror image of routine port. With this port placement and primary surgeon positioning we found that surgery was performed in the same time with less visual disorientation and ergonomic difficulties. The complicated tasks of suturing during cruruoplasty and fundoplication were performed without any difficulties.

## CONCLUSION

With increasing number of laparoscopic procedures, it is very likely for surgeons to encounter anatomic variations like SIT. Laparoscopic Nissen fundoplication (LNF) can be performed in SIT safely. As with any laparoscopic procedure, port positioning and primary surgeon position is critical for performance of laparoscopic surgery in these patients. Gastroesophageal junction being closer to midline, visual disorientation is less during fundoplication than laparoscopic cholecystectomies in SIT. Primary surgeon position in between the legs of the patient provides least visual disorientation and this position is ergonomically more suitable to overcome the technical problems. We strongly believe that proper positioning of ports and primary surgeon is of utmost importance to overcome the technical and ergonomic difficulties associated with upper gastrointestinal laparoscopic procedures in SIT.
